# L-Type Calcium Channel Inhibition Contributes to the Proarrhythmic Effects of Aconitine in Human Cardiomyocytes

**DOI:** 10.1371/journal.pone.0168435

**Published:** 2017-01-05

**Authors:** Jianjun Wu, Xiangchong Wang, Ying Ying Chung, Cai Hong Koh, Zhenfeng Liu, Huicai Guo, Qiang Yuan, Chuan Wang, Suwen Su, Heming Wei

**Affiliations:** 1 National Heart Research Institute Singapore, National Heart Centre Singapore, Singapore; 2 Department of Pharmacology, Hebei Medical University, Shijiazhuang, Hebei, China; 3 Department of Toxicology, Hebei Medical University, Shijiazhuang, Hebei, China; 4 Neuroscience & Behavioral Disorders Program, Duke-NUS Medical School Singapore, Singapore; 5 Cardiovascular & Metabolic Disorders Program, Duke-NUS Medical School Singapore, Singapore; Indiana University School of Medicine, UNITED STATES

## Abstract

Aconitine (ACO) is well-known for causing lethal ventricular tachyarrhythmias. While cardiac Na^+^ channel opening during repolarization has long been documented in animal cardiac myocytes, the cellular effects and mechanism of ACO in human remain unexplored. This study aimed to assess the proarrhythmic effects of ACO in human induced pluripotent stem cell-derived cardiomyocytes (hiPSC-CMs). ACO concentration-dependently (0.3 ~ 3.0 μM) shortened the action potentials (AP) durations (APD) in ventricular-like hiPSC-CMs by > 40% and induced delayed after-depolarization. Laser-scanning confocal calcium imaging analysis showed that ACO decreased the duration and amplitude of [Ca^2+^]_i_ transients and increased in the beating frequencies by over 60%. Moreover, ACO was found to markedly reduce the L-type calcium channel (LTCC) currents (I_Ca,L_) in hiPSC-CMs associated with a positive-shift of activation and a negative shift of inactivation. ACO failed to alter the peak and late Na^+^ currents (I_Na_) in hiPSC-CMs while it drastically increased the late I_Na_ in Guinea-pig ventricular myocytes associated with enhanced activation/delayed inactivation of I_Na_ at -55 mV~ -85 mV. Further, the effects of ACO on I_Ca,L_, I_Na_ and the rapid delayed rectifier potassium current (I_kr_) were validated in heterologous expression systems by automated voltage-clamping assays and a moderate suppression of I_kr_ was observed in addition to concentration-dependent I_Ca,L_ inhibition. Lastly, increased beating frequency, decreased Ca^2+^ wave and shortened field potential duration were recorded from hiPSC-CMs by microelectrode arrays assay. In summary, our data demonstrated that LTCC inhibition could play a main role in the proarrhythmic action of ACO in human cardiomyocytes.

## Introduction

Aconitum is a well-known medical herb which has been used world widely for over 2000 years. However, aconitine (ACO), the main effective ingredient of aconitum and a highly toxic diterpenoid alkaloid, has long been associated with severe cardiovascular toxicities including tachyarrhythmia and hypotension that causes a high mortality in patients [1,2].

Previous studies indicate that ACO is capable of inducing ventricular tachycardia (VT) and ventricular fibrillation (VF) in mouse [3], rat [4], Guinea-pig [5] and rabbits [6]. In isolated sheep heart Purkinje fibers, ACO has been shown to act as an cardiac Na^+^ channel agonist that opens the Na^+^ channels during the depolarization/repolarization phase of an action potentials (AP), leading to a delayed repolarization and early after-depolarization [7]. Similar effects of ACO were found in isolated ventricular myocytes of mice [8] and rats [9] and potentially in Guinea-pigs as well [10].

In addition, ACO-induced L-type Calcium channel (LTCC) inhibition has been observed in neonatal [11] and isolated adult [12] rat cardiac myocytes. Accumulated intracellular Na^+^ has been recognized for activating the reverse-mode of Na^+^-Ca^2+^ exchanger (NCX) and increasing cytosolic [Ca^2+^]_i_, which can trigger intercellular [Ca^2+^]_i_ concentration dependent inactivation (CDI) of the LTCC [13,14]. It remains unclear if LTCC inhibition by ACO is due to accumulation of [Na^+^]_i_.

There are other possible mechanisms involved in ACO-induced toxicity. ACO has also been found capable of blocking the Kv1.5 channels that mediate the outward ultra-rapidly activating delayed rectifier K^+^ current (I_Kur_) in neonatal rat ventricular myocytes [15,16] while studies also demonstrated that ACO inhibited hERG in Xenopus laevis oocytes [16] and HEK293 cells [17].

Despite many intoxication incidences occurred each year, the proarrhythmic effects of ACO in human and the underlying mechanism remain unexplored due to a lack of human cardiomyocyte models. The recent scientific breakthrough has enabled the generation of human induced pluripotent stem cells (hiPSCs)-derived cardiomyocytes (hiPSC-CMs) which share important structural and functional similarities with native human cardiac myocytes. To date, hiPSC-CMs have been successfully adopted for modeling various cardiac diseases [18,19] and for drug testing [20,21].

We hypothesize that ACO-induced arrhythmia in human may involve an alternative mechanism different from that observed in animals due to a marked inter-species variation in cardiac electrophysiology [22,23].

In the present studies, we showed that ACO is able to block the I_Ca,L_ and calcium-induced calcium release (CICR) in hiPSC-CMs without altering I_Na_. Such effects could contribute to the shortened repolarization period characterized by shortened AP duration (APD) and field potential duration (FPDc).

## Materials and Methods

### Chemicals and drugs

ACO, Bay K-8644, and nifedipine were obtained from Sigma-Aldrich (St. Louis, USA). Tetrodotoxin (TTX) was obtained from Aik Moh (Singapore). The stock solutions of ACO (25 mM), Bay K-8644 (1 mM) and nifedipine (1 mM) were prepared in ethanol and stored at 4°C. The stock solution of TTX (5 mM) was prepared in extracellular solution and stored at -80°C.

### Conventional patch-clamp recordings of cardiomyocytes

Cardiomyocytes used in this study were hiPSC-CMs purchased from Cellular Dynamics International Inc (Madison, USA). Cells were maintained at 37°C in a humidified CO_2_ (5%) incubator in maintenance medium provided by the company.

Whole cell configuration of the patch-clamp technique was used to measure action potentials (AP) and calcium and sodium currents. The signal was amplified using an Axon 700B patch-clamp amplifier (Axon Instrument, Sunnyvale, USA) and low-pass filtered at 5 kHz. Patch pipettes were fabricated from glass capillaries (O.D, 1.5mm; I.D, 0.9mm) using a Sutter P-97 microelectrode puller (Novato, CA, USA) and the tips were heat polished with a microforge (NARISHIGE MF-900) to gain a resistance of 2–4 MΩ. The electrical signals were sampled at 2.5–10 kHz and filtered at 2 kHz using a low-pass filter. Data acquisition was achieved using the Digidata 1440A (Axon Instrument). Data analysis and fit were performed using clamp fit 10.2 (Axon Instrument) and Origin 7.0 software (Origin Lab Corporation). A pClamp software (Version8.1; Axcon Instrument) was used to generate voltage-pulse protocols, acquire and analyze data.

#### Action potential recordings in hiPSC-CMs

The APs were recorded under current-clamp mode in normal Tyrode’s solution contained (in mM): NaCl 140, KCl 5.4, CaCl_2_ 1.8, MgCl_2_ 1, glucose 10, and HEPES 10, adjusted to pH 7.4 with NaOH. Pipette solution contained (in mM): KCl 130, NaCl 5, MgCl_2_ 1, MgATP 3, EGTA 10, and HEPES 10, adjusted to pH 7.2 with KOH. The parameters of APs include AP durations (APD) at 30%, 50% and 90% of repolarization (APD_30_, APD_50_, and APD_90_), AP amplitude (APA), maximal diastolic potential (MDP), maximal upstroke velocity (dV/dt_Max_) and beating frequency (BF) were analyzed [18,19]. The APDs were corrected by heart rates with Fridericia’s formula (APDc = APD/interspike interval^1/3^) [24]. Cells were maintained at 35°C by a temperature controller (Warner Instruments, Hamden, USA) during the recording of action potential.

#### L-type calcium current (I_Ca,L_) recording in hiPSC-CMs

Patch pipette solution contained (in mM): CsCl 120, MgCl_2_ 3, MgATP 5, EGTA 10, and HEPES 5, adjusted to pH 7.2 with CsOH. External solution contained (in mM): NaCl 140, CsCl 10, CaCl_2_ 1.8, MgCl_2_ 1, glucose 10, and HEPES 10, adjusted to pH 7.4 with NaOH. To eliminate the ‘run-down’ effect during I_Ca,L_ recordings, Ba^2+^ was also used in the external solution (BaCl_2_ 1.8 mM) to replace Ca^2+^ as charge carrier of calcium channel current [25]. Current-Voltage curve were generated by voltage-clamp protocols consisting of V_hold_ = -80 mV followed by a 3s long pre-pulse at -50 mV to inactivate Na^+^ and T-type Ca^2+^ channels, then a family of 300 ms depolarization from -50 mV to 50 mV in 10 mV increments.

Calcium channel current densities were obtained by dividing current amplitudes by membrane capacitances. Steady state (SS) inactivation variables of I_Ca,L_ were determined using a two-pulse gapped protocol. Potential was held at -40 mV, then pulsed to a conditioning pre-pulse ranging from -80mV to +10mV for 2000 ms, returned to -40 mV for 10 ms, and stepped to 0 mV for 250 ms at 10 s intervals. Voltage-dependence of activation curve and SS-inactivation curve were fitted with Boltzman equation (G = Gmax×[1+exp(V_1/2_-V)/κ]^-1^), where G is the conductance at various test potentials and was calculated from the peak current according to G = I/(V-Vrev), Vrev is the reversal potential obtained by extrapolating the linear part of the I/V curve to its intersection with the voltage axis. Gmax is maximum conductance; V_1/2_ and κ are half-activation voltage and the slope factor. The concentration-response data were fitted with Hill equation: I/Imax = 1/[1+(D/IC_50_)^n^], where I is the peak current in various concentrations of compound, Imax is the maximal peak current, D is the compound concentration, IC_50_ is the drug concentration for 50% inhibition, and n is the Hill coefficient.

The time course of recovery from inactivation of I_Ca,L_ was studied using a two-pulse protocol: a 250-ms pre-pulse (P1) at 0 mV from the holding potential of -50 mV followed by a variable recovery period and a 250-ms test pulse (P2) at 0 mV to assess the amount of current recovered. Each two-pulse sequence was separated by a 30 s interval. The time course of recovery for I_Ca,L_ was determined by fitting the data points to a single exponential function: I/Imax = 1-exp(-t/τ), where Imax and I were the peak current at pre-pulse (P1) and test pulse (P2), respectively; t was the variable recovery time; τ was the recovery time constant.

#### Peak and late sodium current (I_Na_ and I_NaL_) recording in hiPSC-CMs and Guinea-pig ventricular myocytes

See S1 File (Supplemental Methods).

### I_Na_, I_Kr_ and I_Ca,L_ recording in the heterologous expression systems by automated patch-clamping technique

See S1 File (Supplemental Methods) [26].

### Laser-scanning confocal calcium imaging

[Ca^2+^]_i_ transients were recorded in hiPSC-CMs using a LSM-710 laser-scanning confocal microscope (Carl Zeiss, Inc, Germany) with a 40×, 1.3 numerical aperture oil immersion objective and axial resolutions of 1.5 μm. Briefly, hiPSC-CMs were loaded with 2 μM Fluo-8 AM (AAT Bioquest, Inc. Sunnyvale, CA, USA) for 15 min at 37°C, and recorded in normal Tyrode’s solution. Fluo-8 was excited at 488 nm, and fluorescence emission was measured at 505 nm. Images were acquired in the line-scan (X-T) mode with 512 pixels (pixel intervals of 0.15 μm) per line at a rate of 3 ms per scan. The [Ca^2+^]_i_ transients were analyzed using a modified version of MATLAB program. The Ca^2+^ fluorescence emission intensity was expressed as F/F_0_ where the F_0_ was the basal fluorescence intensity level. The recording was performed at 35°C temperature.

### The microelectrode arrays (MEA) assay on hiPSC-CMs

See S1 File (Supplemental Methods) [24].

### Statistical analysis

Values presented are means ± standard error of means (SEM) for electrophysiology assays and means ± standard derivation (SD) for calcium imaging assay. Statistical comparisons were made using Student’s unpaired or paired *t*-test. A value of p<0.05 was considered statistically significant.

## Results

The current study adopted concentrations of ACO ranged from 0.03 to 3 μM which is consistent with that (0.1 ~10 μM) adopted in the previous in vitro studies [7~10] and it covered the blood concentrations (0.02 ~ 0.11 μM) identified in patients with ACO-induced lethal cardiac arrhythmia [27].

### Effects of ACO on APs of hiPSC-CMs

**Fig 1:**
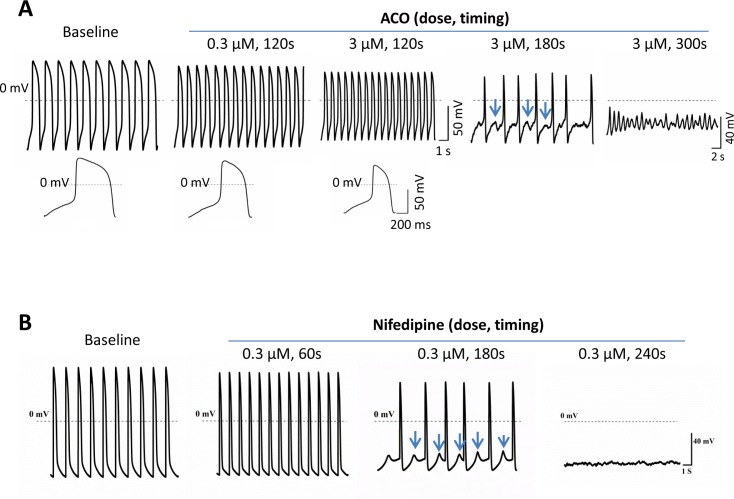
Effects of ACO on action potentials of hiPSC-CMs. (A) Representative AP waveforms recorded in a hiPSC-CM with 120 seconds (s) exposure of ACO at 0.3 and 3.0 μM and 180s and 300s exposure of ACO at 3.0 μM. Increased beating frequencies and decreased APD and APA were observed with short-term (120s) ACO treatment. Prolonged treatment of ACO at 3.0 μM led to frequent DADs and rapid irregular VF-like AP changes. (B),Representative AP waveforms recorded in a hiPSC-CM exposed to 0.3 μM nifedipine for 60s ~ 240s. Increased beating frequencies and decreased APD occurred at 60s while DADs emerged at 180s. APs were suppressed at 240s. The arrows indicate DADs.

Effects of ACO on APs were measured in ventricular (V)-like hiPSC-CMs which were characterized based on their AP properties including APD, APA and dV/dt_Max_ [18,19,28]. ACO (0.3, 1.0 and 3.0 μM) concentration-dependently increased the BF, shortened APD30, APD50 and ADP90, and reduced APA in hiPSC-CMs while prolonged exposure (180~300 seconds) to a higher concentration of ACO (3.0 μM) led to more remarkable effects including a positively shift of the MDP, increased prevalence of DADs followed by VF-like AP waveforms till their complete diminishment (Fig 1A, Table 1, data for ACO at 1.0 μM not shown). The effects of ACO resembled that of nifedipine, a specific LTCC blocker. Nifedipine (0.3 μM) increased beating frequency, shortened APDs and triggered DADs in hiPSC-CMs followed by a complete abolishing of APs (Fig 1B).

**Table 1:** Effects of ACO on action potentials of hiPSC-CMs.

hiPSC-CMs (n = 7)	Baseline	ACO (0.3μM)	ACO (3μM)
APA (mV)	110.92 ± 1.78	103.42 ± 2.42*	96.31 ± 1.32^†^
APD30 (ms)	402.46 ± 47.98	316.40 ± 35.60*	199.22 ± 16.89^†^
APD50 (ms)	456.03 ± 50.89	370.81 ± 38.79*	237.43 ± 16.92^†^
APD90 (ms)	520.77 ± 58.28	436.45 ± 39.62*	296.42 ± 16.06^†^
APD90/APD30	1.305 ± 0.04	1.401 ± 0.06	1.518 ± 0.06^†^
APD90/APD50	1.14 ± 0.02	1.19 ± 0.02^†^	1.26 ± 0.03^‡^
MDP (mV)	-62.10 ± 1.49	-60.75 ± 1.35	-59.68 ± 1.14*
Over shoot (mV)	48.82± 1.67	42.67 ± 2.40*	36.64 ± 1.78^†^
BF (beats/min)	48.60 ± 4.16	67.35 ± 7.79*	77.83 ± 8.08*
No. of DAD/5 min	0	13.25±3.87^†^	60.16±4.35^†^

All the parameters were measured in the ventricular-like hiPSC-CMs exposed to ACO for 120 seconds. APD_30_, APD_50,_ andAPD_90,_ action potential duration at 30%, 50% and 90% of repolarization (APDs are corrected by beating rates). APA, action potential amplitude; MDP, maximal diastolic potential; BF, beating frequency; DAD, delayed after-depolarization. Results are shown as Mean ± SEM.

* *P*<0.05

^†^ *P*<0.01

^‡^ *P*<0.001 (vs. baseline).

Our data demonstrated nifedipine-like effects of ACO in hiPSC-CMs characterized by shortening of APDs followed by DAD and VF-like changes.

### Effects of ACO on [Ca^2+^]_i_ transients in hiPSC-CMs

**Fig 2:**
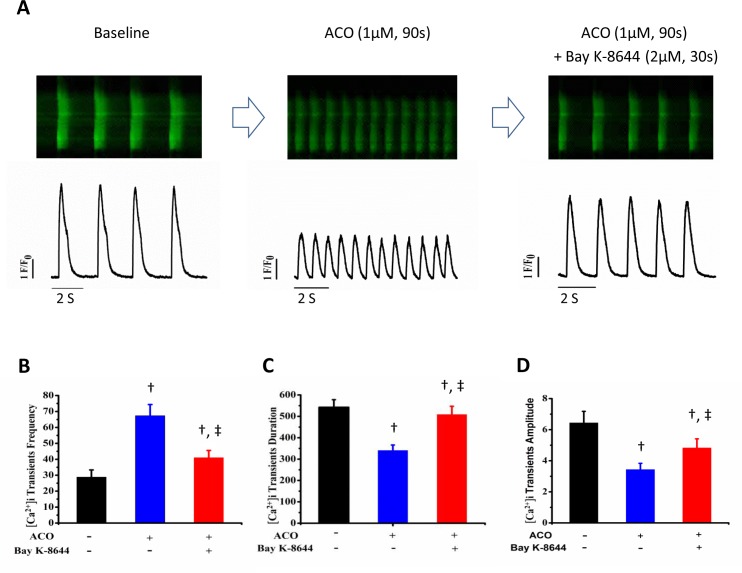
Effects of ACO on [Ca^2+^]_i_ transients in hiPSC-CMs. (A) Representative traces of the [Ca^2+^]_i_ transients recorded in a hiPSC-CM at baseline and with subsequent exposure of ACO and ACO plus Bay K-8644. (B, C, and D) Bar-graphs show the changes in [Ca^2+^]_i_ transient frequency, duration and amplitude in hiPSC-CMs. ^†^*p* < 0.01, vs. baseline. ^‡^ *p* < 0.001, vs. ACO. n = 7.

In hiPSC-CMs, [Ca^2+^]_i_ transients reflect calcium-induced calcium release (CICR) triggered by I_Ca,L_ influx [29] and closely correlate with action potentials [30,31]. To validate the nifedipine-like effects of ACO on APs, [Ca^2+^]_i_ transients were recorded in hiPSC-CMs. ACO at 0.3, 1.0 and 3.0 μM increased the frequency and decreased the duration and amplitude of [Ca^2+^]_i_ transients and such effects were partially abolished by Bay K-8644, a LTCC opener. Fig 2 demonstrates the effects of ACO at 1.0 μM. Long time (180~300 seconds) exposure of ACO at 3.0 μM completely abolished the [Ca^2+^]_i_ transients (data not shown).

Data from [Ca^2+^]_i_ transient assay further consolidated that the effects of ACO on APs are most likely to be achieved through inhibition of LTCC and CICR.

### Effects of ACO on I_Na_ and I_Ca,L_ in hiPSC-CMs

Our data of AP and [Ca^2+^]_i_ transients indicated that LTCC inhibition by ACO could be a major ionic change in human cardiomyocytes, which is different from the cardiac Na^+^ channel activation observed in animal cardiac myocytes. To validate the changes in cardiac ion currents by ACO, I_Na_ and I_Ca,L_ were measured in ACO-treated hiPSC-CMs.

#### Effects of ACO on I_Na_ in hiPSC-CMs

**Fig 3:**
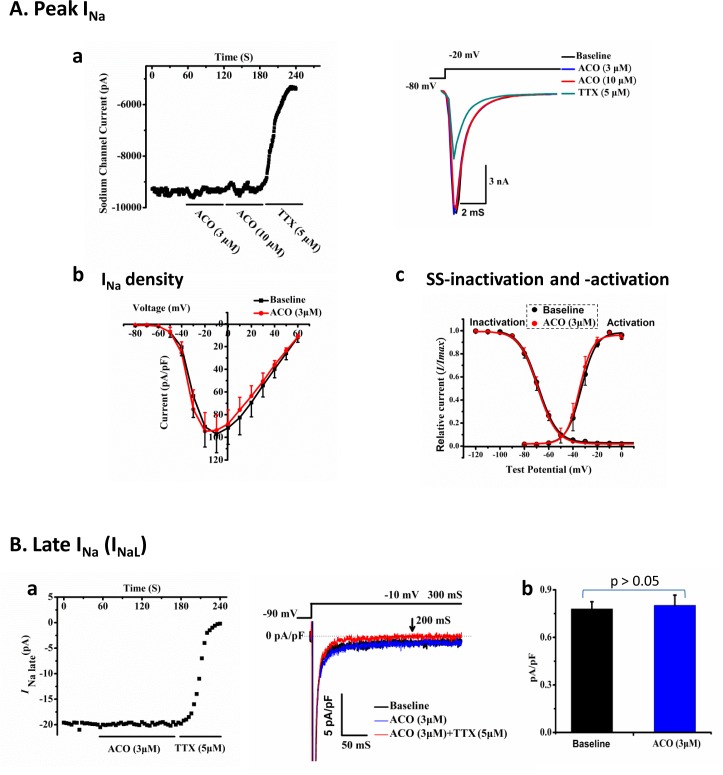
Effects of ACO on sodium currents in hiPSC-CMs. (Aa) A representative time course and corresponding traces of the peak I_Na_ recorded in a hiPSC-CM showing the effect of ACO and TTX. (Ab) The I-V curve showing peak I_Na_ density in hiPSC-CMs. n = 7. (Ac) The SS-inactivation and -activation curves. n = 7. (Ba) A representative time course and the corresponding traces of late I_Na_ (I_NaL_) recorded in a hiPSC-CM showing the effects of ACO and TTX. (Bb) A bar-graph showing the average densities of the I_NaL_ (at 200ms) recorded in hiPSC-CMs treated with 3 μM ACO. n = 7.

The peak I_Na_ and late I_Na_ (I_NaL_) were measured. ACO of up to10.0 μM exerted no significant effects on the density of I_Na_ (Fig 3Aa and 3Ab). Neither did ACO alter the SS-activation and -inactivation of I_Na_ (Fig 3Ac, Table 2) which also reflect the window currents. Moreover, ACO failed to alter I_NaL_ (Fig 3B).

**Table 2:** Effect of ACO on SS-activation and -inactivation of I_Na_ in hiPSC-CMs.

		Baseline	ACO (3 μM)	*P* value
SS- activation (n = 7)	V_1/2_	-31.13 ± 1.24	-31.58 ± 2.36	0.87
	к	4.32 ± 0.50	4.31 ± 0.77	0.99
SS- inactivation (n = 7)	V_1/2_	-70.68 ± 2.03	-70.48 ± 1.35	0.93
	к	6.66 ± 0.57	6.40 ± 0.48	0.74

V_1/2_, half-activation/inactivation voltage. К, slope factor. Results are shown as Mean ± SEM.

*P* values were calculated by comparing ACO with baseline.

#### Effects of ACO on I_Ca,L_ in hiPSC-CMs

**Fig 4:**
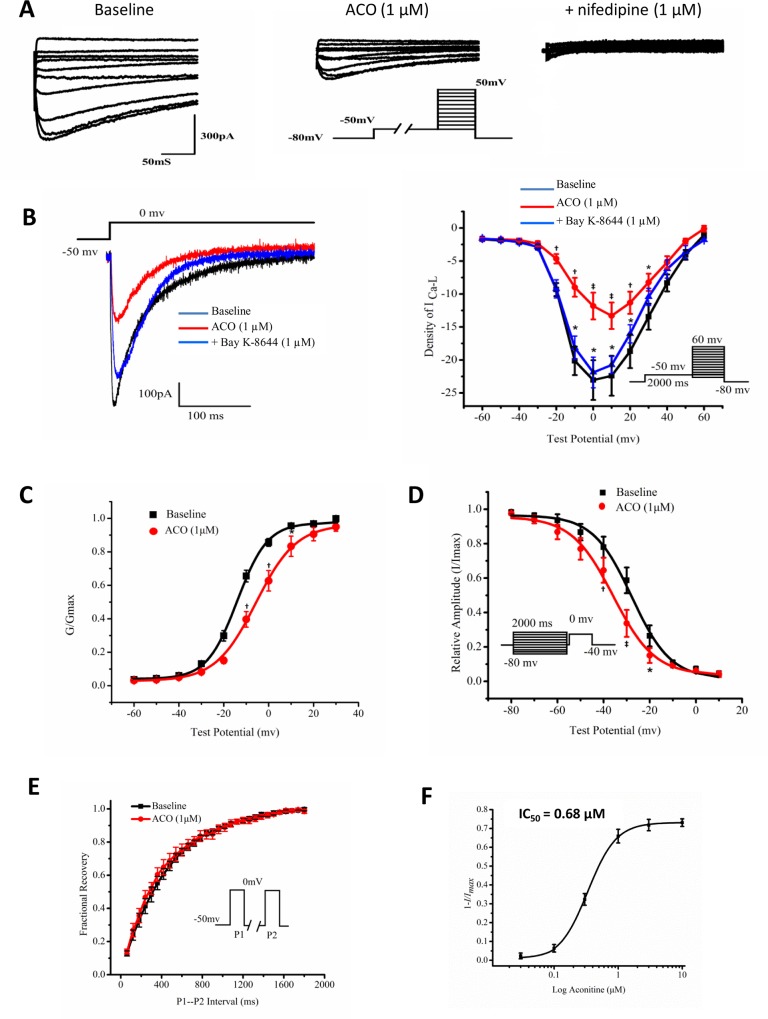
Effects of ACO on I_Ca,L_ in hiPSC-CMs. (A) Representative traces of the voltage-gated LTCC current I_Ca,L_ recorded from a hiPSC-CM at baseline followed by exposure of 1 μM ACO and addition of 1 μM nifedipine. (B) Representative traces and I-V curves recorded from hiPSC-CMs exposed to 1 μM ACO followed by 1 μM Bay K-8644. *p < 0.05, ^†^*p* < 0.01, ^‡^ *p* < 0.001, ACO vs. baseline or Bay K-8644 vs ACO, n = 8. (C) The voltage-dependent activation curve of I_Ca,L_ recorded in hiPSC-CMs exposed to 1 μM of ACO. G/G_max_: normalized peak conductance. *p < 0.05, ^†^*p* < 0.01, vs. baseline. n = 5. (D) The SS-inactivation curve of I_Ca,L._ *p < 0.05, ^†^*p* < 0.01, ^‡^ *p* < 0.001, vs. baseline. n = 5. (E) Recovery from inactivation of I_Ca,L_. n = 5. Insert shows the 2-pulse protocol. (F) The IC_50_ of ACO on I_Ca,L_ (n = 8).

With the “run-down” phenomenon minimized after replacement of extracellular Ca^2+^ with Ba^2+^, the inhibitory effect of ACO on I_Ca,L_ was confirmed and validated by addition of nifedipine which further blocked I_Ca,L_ (Fig 4A).The inhibitory effect of ACO on LTCC was further validated by addition of Bay K-8644 (1.0 μM), an I_Ca,L_ opener, which reversed the effect of ACO (Fig 4B). Moreover, a positive shift of the half-activation voltage (V_1/2_) and a negative shift of the V_1/2_ of I_Ca,L_ were observed in hiPSC-CMs treated with ACO while the recovery from inactivation remained unchanged (Fig 4C, 4D and 4E, Table 3). hiPSC-CMs exposed to 0.1 ~ 10.0 μM of ACO demonstrated a strong concentration-dependent inhibitory effect on I_Ca,L_ with an IC_50_ of 0.68 ± 0.15 μM (Fig 4F).

**Table 3:** Effect of ACO on the kinetics of I_Ca,L_

		Baseline	ACO (1 μM)	*P* value
Activation (n = 8)	V_1/2_	-13.81 ± 0.69	-5.66 ± 1.27	**0.010**
	к	6.94 ± 0.53	8.25 ± 0.72	0.082
SS-inactivation (n = 8)	V_1/2_	-27.69 ± 1.43	-34.06 ± 1.69	**0.016**
	к	8.94 ± 0.87	10.6 ± 1.02	0.371
Recovery from Inactivation (n = 5)	τ	441.45 ± 35.13	413.12 ± 44.99	0.667

V_1/2_, half-activation/inactivation voltage. К, slope factor. τ, the recovery time constant. Results are shown as Mean ± SEM. *P* values were calculated by comparing ACO with baseline.

### Effects of ACO on I_Na_, I_Kr_ and I_Ca,L_ recorded in the heterologous expression system

**Fig 5:**
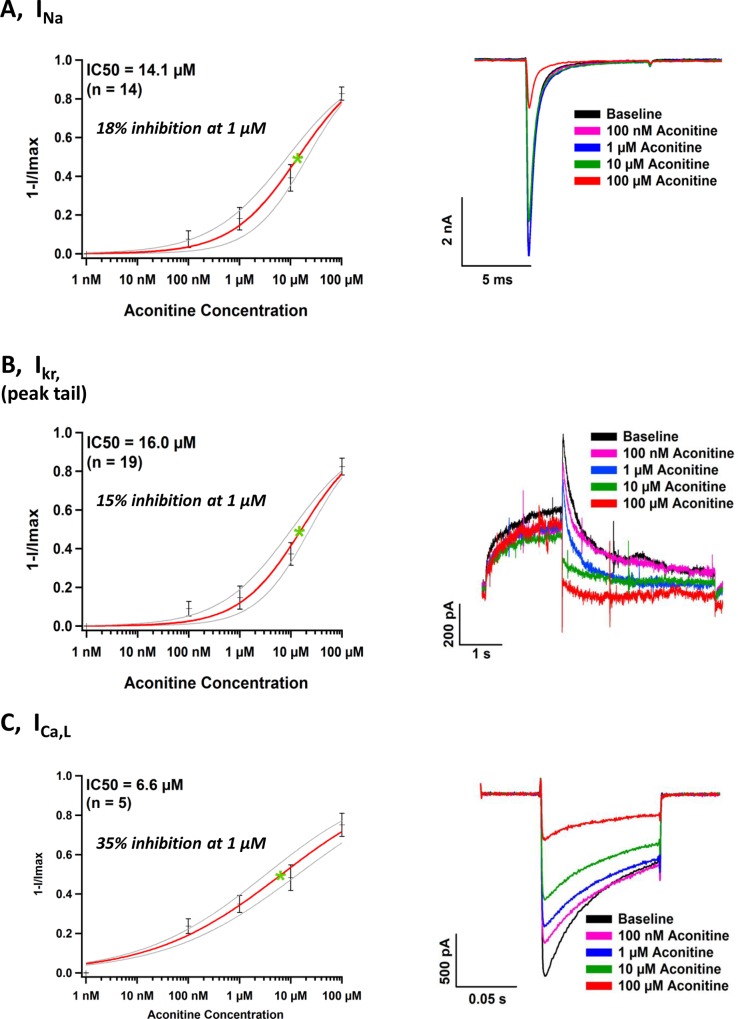
Effects of ACO on I_Na_, I_Kr_ and I_Ca,L_ in heterologous expression systems. Nav1.5-HEK293, hERG-HEK293 and Cav1.2-CHO cells were exposed to 0.1~100 μM ACO. (A) The concentration-dependent inhibition curve of I_Na_ and representative traces recorded. (B) The concentration-dependent inhibition curve of I_Kr_ (peak tail: the peak of the tail current) and representative traces. (C) The concentration-dependent inhibition curve of I_Ca,L_ and representative traces.

Effects of ACO on major ion currents in hiPSC-CMs were validated in the heterologous expression systems. Using the Patchliner® automated patch-clamping system, effects of ACO (0.01 ~100 μM) on I_Na_, I_Ca,L_ I_Kr_ were measured in Nav1.5-HEK293, Cav1.2-CHO and hERG-HEK293 cells, respectively. ACO showed milder blockage of I_Na_ and I_Kr_ in Nav1.5-HEK293 and hERG-HEK293 cells with IC_50s_ of 14.1 μM (n = 14) and 16.0 μM (n = 19), respectively (Fig 5A and 5B). However, a stronger I_Ca,L_ blocking was noted in Cav1.2-CHO cells exposed to lower concentrations of ACO with IC_50_ of 6.6 μM (n = 5) (Fig 5C).

### Effects of ACO on extracellular filed potentials

**Fig 6:**
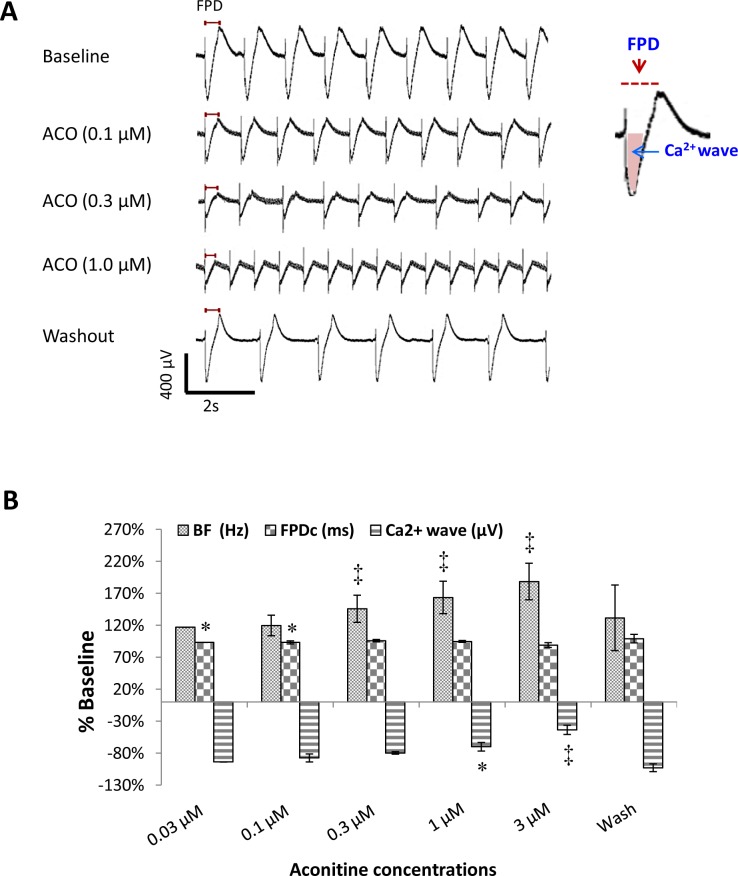
Effects of ACO on the field potentials in hiPSC-CMs. (A) Representative traces of the filed potentials (FD) recorded with a cluster of hiPSC-CMs at baseline, exposed to various concentrations of ACO and after washout. (B) A bar-graph demonstrates the average beating frequency (BF), Ca^2+^ waves and FPDc recorded at baseline, with 0.03, 0.1, 0.3, 1.0 and 3.0 μM of ACO treatments and after washout. *p< 0.05, ^‡^*p*< 0.001; vs. baseline. n = 4.

To validate the potential impact of ACO-induced APD shortening on electrocardiography (ECG), the extracellular field potentials (FP) of hiPSC-CMs were measured using the MEA system which produces signals resemble the ECG. ACO-treated hiPSC-CMs showed a tendency of concentration-dependent increase of BF, decrease of Ca^2+^ wave and shortening of FPDc (Fig 6). Yet the heart rate increments and Ca^2+^ wave reduction were obvious at higher concentrations of ACO while a moderate FPDc shortening was significant at 0.03 and 0.1 μM of ACO (Fig 6B). Cell contractions were stopped by ACO at higher concentrations and with longer exposure time (> 3 μM and > 300s) (data not shown).

Data from the MEA assay, including the increased BF, decreased Ca^2+^ wave (which echoes I_Ca,L_ inhibition observed with single cells) and shortened FPDc, support our findings in single hiPSC-CM.

### Effects of ACO on I_Na_ in isolated Guinea-pig ventricular myocytes

**Fig 7:**
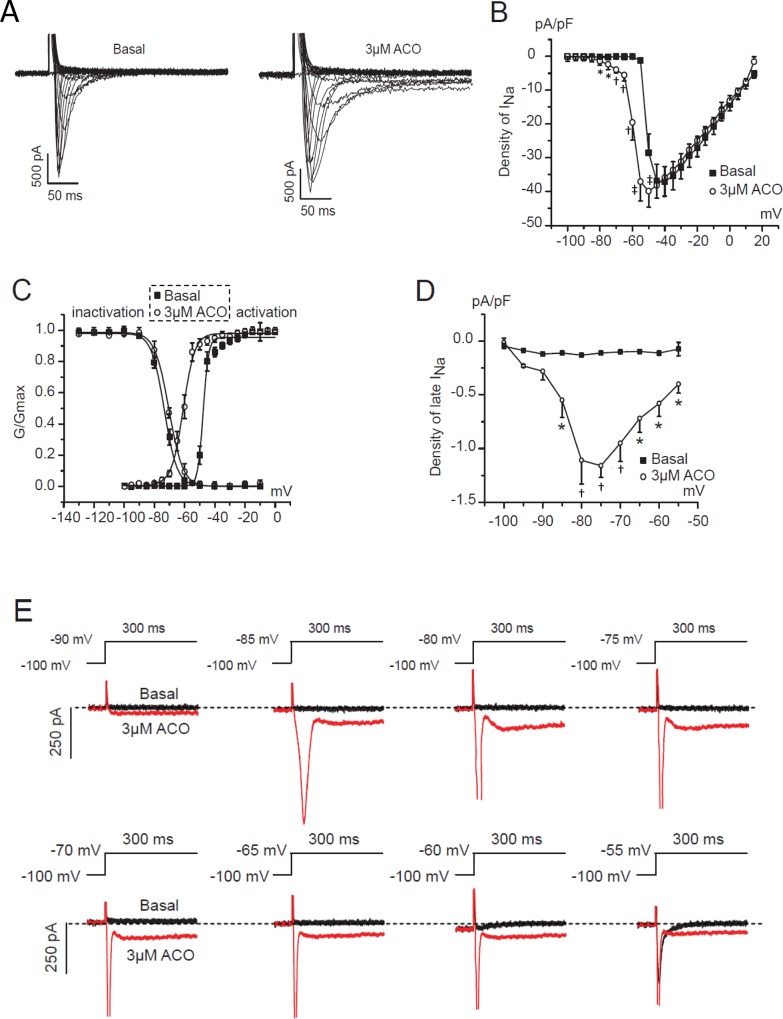
Effects of ACO on sodium currents in Guinea-pig ventricular myocytes. (A) Representative traces of the peak I_Na_ recorded in a Guinea-pig ventricular myocyte prior to and after 3 μM ACO. (B) The I-V curves showing the current densities of peak I_Na_. n = 5. (C) The SS-inactivation and -activation curves. n = 5. (D) The I-V curves of the voltage-gated I_NaL_ densities. n = 5. (E) Representative traces of voltage-dependent I_NaL_ recorded in a Guinea-pig ventricular myocyte prior to and post 3μM ACO. *p < 0.05, ^†^*p* < 0.01, vs. baseline.

To validate the effect of ACO on sodium currents in hiPSC-CMs, I_Na_ and I_NaL_ were measured in isolated Guinea-pig ventricular myocytes treated with 3 μM ACO. The peak sodium current densities recorded during -80mV to -50mV significantly increased by ACO while it remained unchanged during -45 mV to +20 mV (Fig 7A and 7B). Moreover, ACO negatively shifted the activation of I_Na_ by 12.58 mV (-47.66 ± 0.71 vs -60.24 ± 2.22) and positively shifted the inactivation of I_Na_ by 4.32 mV (-69.97 ± 1.78 vs -74.29 ± 1.20) (Fig 7C, Table 4) and markedly increased the I_NaL_ at the voltages between -55 and -85 mV (Fig 7D and 7E). Thus, the well-anticipated effect of ACO on opening of the I_NaL_ was validated in Guinea pig cardiac myocytes.

**Table 4:** Effect of ACO on SS-activation and -inactivation of I_Na_ in Guinea-pig ventricular myocytes.

		Baseline	ACO (3 μM)
SS-activation (n = 5)	V_1/2_	-47.66 ± 0.71	-60.24 ± 2.22^†^
	к	1.77 ± 0.38	1.10 ± 0.094
SS-inactivation (n = 5)	V_1/2_	-69.97 ± 1.78	-74.29 ± 1.20*
	к	4.03 ± 0.19	3.89 ± 0.22

V_1/2_, half-activation/inactivation voltage. К, slope factor. Results are shown as Mean ± SEM.

* *P*<0.05

^†^ *P*<0.01 (vs. baseline).

## Discussion

In the current study, we observed that ACO, within the well-adopted in vitro doses and closer to the toxicological concentrations, is capable of triggering proarrhythmic effects in human cardiomyocytes characterized by increased beating frequency and shortened repolarization phases of action potentials and field potentials followed by increased prevalence of DADs and cardiac arrest. Such changes were accompanied by reduced duration and amplitude of [Ca^2+^]_i_ transients and Ca^2+^ wave. Moreover, data from hiPSC-CMs and the heterologous expression systems directly indicated that ACO could selectively inhibit I_Ca,L_ independent from [Na^+^]_i_ and Na^+^ channel activities as the peak, late and window I_Na_ remained unaffected.

ACO has long been known as a neurotoxin that selectively interacts with the voltage-dependent neuronal Na^+^ channels by binding to its neurotoxin binding site 2 at the pore region of the alpha subunit, rendering a prolonged opening of the Na^+^ channel [32]. In isolated cardiac myocytes of various animals, ACO has been shown to act as an I_Na_ agonist that opens the Na^+^ channels during the depolarization/repolarization phases, leading to a delayed repolarization [7~9]. However, our data from human hiPSC-CMs and transgenic cell line (Nav1.5-HEK293), controlled by Guinea-pig ventricular myocytes, minimized the possibility that ACO-increased cytosolic [Na^+^]_i_ in human cardiomyocytes acts as the main driver of cardiac arrhythmia [13,14]. Rather, our data indicate that inhibitions of LTCC and CICR by ACO could play a key role in the proarrhythmic effects of ACO in human and ACO is capable of blocking LTCC following a mechanism independent from increased [Na^+^]_i_. The effects of ACO on cardiac repolarization in hiPSC-CMs resemble that of nifedipine, which, like Bay K-8644, belongs to dihydropyridines, the most selective and potent calcium channel modulators specifically identify LTCC activity in cardiac myocyte [33]. Moreover, the effect of ACO on beating frequency also resemble that of nifedipine that has been shown capable of increasing the BF in cardiomyocytes derived from hiPSCs and human embryonic stem cells [34,35].

Defects in cardiac LTCC have been associated with severe arrhythmias in human. Loss-of-function of cardiac LTCC due to genetic mutations have been associated with inherited J-wave syndromes and sudden cardiac death [36] and short QT syndrome characterized by an abnormally short QT interval and an increased risk of atrial and ventricular fibrillation [25,37]. Decreased I_Ca,L_ has been found in patients with atrial fibrillation [38].

Nevertheless, caution should be exercised to apply the data obtained in hiPSC-CMs to the intact human heart. ECG findings in patients with ACO-induced cardiotoxicity have been explained so far by the persistent activation of Na^+^ channels and subsequent accumulation of cytosolic Ca^2+^. The current study, however, suggests that I_Ca,L_ inhibition plays a key role in the effects of ACO in human cardiomyocytes. We noted that ACO at lower concentrations and shorter exposure time (< 3 μM and < 300s) could rapidly shorten the repolarization phase (APD and FPDc) in hiPSC-CMs while increased dosages and longer exposure time abolished the electrical activities (Figs 2A and 6). In patients with aconitine intoxication, I_Ca,L_ inhibition, rather than I_Na_ activation, may offer a new explanation for atrioventricular blocks and bradycardia, the major ECG defects identified in almost all patients, in addition to the effect of parasympathetic activation, as I_Ca,L_ is the major driver of the depolarization of the pacemaker myocytes. However, data from hiPSC-CMs fall short to explain why QT-shortening has not been found in patients yet QT prolongation has been reported in some patients instead [39]. While the neurohormonal effects of ACO in cardiotoxicity [1] may explain some of the discrepancy between the electrophysiological characteristics of a ventricular myocyte (such as APs) and the heart (such as ECG), a possible role of I_Kr_ inhibition, the doses of ACO and the exposure time/duration might all contribute to the variable cardiac repolarization durations in human heart. Firstly, we recorded a moderate I_Kr_ inhibition in hERG-HEK293 cells treated by ACO which is consistent with previous in vitro studies [16,17]. Under certain conditions, the effects of I_Kr_ inhibition may become dominant over that of I_Ca,L_. Secondly, different species of ACO-containing herbs and different ways of administration could contribute to different concentrations of ACO in patients. Lastly, our data from hiPSC-CMs were obtained within 30 minutes while it takes an average of 4 hours (earliest 2 hours) to have the 1^st^ ECG recorded in patients with ACO ingestions [1], leaving the early change of ECG unchecked.

In addition, our data hint that ACO may also inhibit LTCC in vascular smooth muscle cells and compromise the vascular tone and thus contribute to the severe hypotensive effect in addition to its actions on autonomic nervous system. It is reported that overdose of LTCC inhibiters could cause severe hypotension [40] likely due to reduced vascular tones following the LTCC inhibition in vascular smooth muscle cells [41].

Although blocking of Kv1.5 channels and I_Kur_ in ACO-treated neonatal cardiomyocytes have been associated with prolonged APDs and EADs [15,16], such changes are unlikely to happen in human ventricular myocytes and pacemaker cells as I_kur_ is predominantly expressed in atrial myocytes [42].

Aconitum, along or as an important ingredient in many traditional Chinese medicine prescriptions, will continually be adopted for treating various disease conditions and ACO-induced arrhythmias will remain to be a health threat [1,2]. To date, there is no effective ion channel-targeted therapy for ACO poisoning as sodium channel blockers appeared to be the least effective drugs [1]. Our data may shed new light on more targeted drug therapies.

The current study highlights the importance of applying human cardiomyocyte models for evaluating the effects of various drugs. The identification of a different mechanism of ACO-induced arrhythmia in human cardiomyocytes compared to that from other animals, particularly rodents, highlights the intra-species variations in cardiac electrophysiology [22, 23]. Data of the current and previous studies indicate that the cardiac Na^+^ channels of different species could have different sensitive to ACO while the TTX-insensitive Na^+^ channels could be insensitive to ACO as a high concentration of ACO (100 μM) [43] on human Na^+^ channels achieved the similar effect of 3 μM ACO (close to physiological concentrations) on isolated Guinea-pig ventricular myocytes. Whereas lower concentrations of ACO of (3~10 μM) failed to alter human I_Na_.

In summary, for the first time we observed the proarrhythmic effects of ACO in human cardiomyocytes and such effects are likely to be achieved via inhibition of LTCC.

## Supporting Information

S1 File Supplemental Methods. (DOC)
