# Supplementary material for: L-Type Calcium Channel Inhibition Contributes to the Proarrhythmic Effects of Aconitine in Human Cardiomyocytes
Source: PLoS One. 2017 Jan 5;12(1):e0168435. doi: 10.1371/journal.pone.0168435 (PMC5215924; doi:10.1371/journal.pone.0168435)
Supplement: S1 File — (DOC) [file pone.0168435.s001.doc]

L-Type Calcium Channel Inhibition Contributes to the Proarrhythmic Effects of Aconitine in Human Cardiomyocytes

Jianjun Wu, Xiangchong Wang, Ying Ying Chung, Cai Hong Koh, Zhenfeng Liu, Huicai Guo, Qiang Yuan, Chuan Wang, Suwen Su and Heming Wei

**Supporting Information**

**S1 File. Supplemental Methods.**

**Recording of the peak and late sodium currents (I_Na_)**

Whole-cell Na^+^ currents were recorded by patch-clamp technique (Axon 700B, Axon Instrument, Sunnyvale, USA) at room temperature (20-22ºC). Briefly, patch pipettes were fabricated by a P-97 Flaming-Brown micropipette puller (Sutter Instruments) and fire polished using a microforge (MF 830, Narishige, Japan). Pipettes with resistance of 1.5 - 2.5 MΩ were used to facilitate intracellular dialysis of cells with the pipette solution and to minimize voltage errors. Cesium-was used in both internal and external solutions to reduce the gradient for sodium entry and to block any contamination from potassium current [1].

**I_Na_ recording in hiPSC-CMs**

Both of the peak and late cardiac Na_v_1.5 sodium currents (I_Na_ and I_NaL_) were recorded in hiPSC-CMs [2,3]. For I_Na_ and I_NaL_ recording, the patch pipettes were backfilled with a Cs^+^-rich internal solution contained (in mM): CsCl 133, NaCl 3, MgCl_2_ 2, EGTA 10, HEPES 5, TEA-Cl 2, and Na_2_-ATP 4, adjusted to pH 7.2 with CsOH. The external solution contained (in mM): NaCl 135, CsCl 10, MgCl_2_ 1.2, CaCl_2_ 1.8, HEPES 10, glucose 10, and nifedipine 0.05, adjusted to pH 7.4 with CsOH. In addition, 65%-75% of series resistance was adopted. The voltage-dependent activation of sodium current was studied by a family of 100 ms depolarization from -80 mV to +60 mV in 10 mV increments. The voltage dependence of steady state (SS) inactivation was studied using 1000 ms pre-pulses from -120 to 0 mV in 10 mV increments, followed by a 50 ms test pulses to -20 mV. Current-voltage relations for SS-activation and SS-inactivation were determined by fitting a Boltzmann function (I/Imax = [1+exp((V-V_1/2_)/k)]^-1^), yielding the membrane potential of the half -maximal activation (V_1/2_-activation) and inactivation (V_1/2_-inactivation) and the slope factor (κ). I_NaL_ was elicited using a 0.25 Hz train of pulses to -20 mV from -100 mV holding potential. I_NaL_ was estimated as the difference between the traces recorded in the absence and presence of TTX (5 µM) at 200 ms [2].

**I_Na_ recording in isolated guinea-pig ventricular myocytes**

The animal work protocol was approved by the Institutional Animal Care and Use Committee of Hebei Medical University (Shijiazhuang, China). Animals were provided by the university experimental animal center. Ventricular myocytes were enzymatically dissociated from the left ventricles of the hearts of adult male guinea-pigs (250-300g) following the standard protocols with some modification **[**4,5**].** In brief, the explanted hearts were retrogradely perfused on a Langendorff system with a Ca^2+^-free Tyrode's solution contained (in mM): NaCl 140, KCl 5.4, MgCl 1.0, HEPES10, and Glucose10, adjusted to pH 7.4 with NaOH. Five minutes later, the perfusion solution was switched to a Tyrode's solution containing Type Ⅱ collagenase (0.4 mg/mL) and the hearts were continually perfused for 12-15 minutes. Next, cardiac myocytes were immediately harvested from the left ventricles of the hearts. The isolated cardiac myocytes were used for patch-clamp recording within 6~8 hours.

I_Na_ and I_NaL_ were recorded following the protocol of Yuill *et al* with some modification [1]. The patch pipettes were filled with a solution contained (in mM): CsCl 120, MgCl_2_ 5, CaCl_2_ 1, Na_2_ATP 5, EGTA 11, and HEPES 10, adjusted to pH 7.4 with CsOH. The external solution contained (in mM): Choline Chloride130, CsCl 5.4, CaCl_2_ 1, MgCl_2_ 1, NaH_2_PO_4_ 0.33, HEPES 10, NaCl 20, and Nimodipine 0.01, adjusted to pH 7.3 with CsOH. To determine the voltage-dependence of steady-state activation, currents were elicited by a 300 ms pulse from a holding potential of -100mV to test potential between -100 mV and 15 mV in 5 mV increments. The sodium conductance (G) was calculated by dividing the peak current for each voltage step by driving force (V_m_-V_rev_) then normalized to the peak conductance (G_max_). Data were fitted with the Boltzmann relationship, G/*G*_max_=1/{1+exp[(*V*_1/2_-*V*_m_)/*k*]} in which V_1/2_ is the voltage at which half of Na^+^ channels are activated, *k* is the slope factor and V_m_ is the membrane potential. Standard two-pulse protocols were used to generate the steady-state inactivation curves from the holding potential of -100 mV. Cells were stepped to 300 ms pre-conditioning potentials varying between -130 mV and -10 mV (pre-pulse), followed by a test pulse to -30 mV. Conductance (G) was normalized to G_max_ and fitted to a Boltzmann function of the from G/*G*_max_=1/{1+exp[(*V*_1/2_-*V*_m_)/*k*]} in which V_1/2_ is the voltage at which half of Na^+^ channels are inactivated, *k* is the slope factor and V_m_ is the membrane potential. Curve fitting and data analysis were performed using Clampfit 10.2 software (Axon Instruments) and Origin 8 (Originlab Corporation). I_NaL_ was recorded with 300 ms voltage pulses gradually stepped from a holding potential of -100 mV to 15 mV in 5 mV increments, then the amplitudes of I_NaL_ were measured at 100 ms before and after ACO application [6].

**I_Na_, I_Kr_ and I_Ca,L_ currents recording in the heterologous expression systems**

Effects of ACO on the voltage-gated fast sodium current (I_Na_), the rapid component of the outward rectifier potassium current (I_Kr_) and LTCC current (I_Ca,L_) were measured in Nav1.5-HEK293 (Anaxon AG, Berne, Switzerland), hERG-HEK293 cells (hERG with β1 Subunit) from Chan Test (Catalog no.CT6001, Cleveland, USA.) and Cav1.2-CHO (hCav1.2/β2/α2δ channel) from ChanTest (Catalog no. CT4004, Cleveland, USA) heterologously expressing human *SCN5A*, *KCNH2* and *CACNA1C* which encodes human Nav1.5, Kv11.1 and Cav1.2, respectively.

I_Na_, I_Kr_ and I_Ca,L_ were measured by Patchliner® automated patch-clamping system (Nanion Technologies, Munich, Germany) [7]. The internal solution for measuring I_Na_ and I_Ca,L_ contained (in mM): CsCl 50, NaCl 10, Cs-Fluoride 60, EGTA 20, and HEPES 10, adjusted to pH 7.2 with CsOH. To prevent rundown when recording calcium channels, Na_3_GTP 0.3, ATP (Mg salt) 5 and BAPTA (free acid) 5, all in mM, were added into the I_Ca,L_ internal solution and adjusted to pH 7.2 with CsOH. The internal solution for measuring I_Kr_ contained (in mM): KCl 50, NaCl 10, K-Fluoride 60, EGTA 20, and HEPES 10, adjusted to pH 7.2 with KOH. The external solution for measuring I_Kr_ and I_Ca,L_ contained (in mM): NaCl 140, KCl 4, MgCl_2_ 1, CaCl_2_ 2, glucose monohydrate 5, and HEPES 10, adjusted to pH 7.4 with NaOH. The external solution for measuring I_Na_ contained (in mM): NaCl 80, KCl 4, MgCl_2_ 1, CaCl_2_ 2, glucose monohydrate 5, NMDG 60 and HEPES 10, adjusted to pH 7.4 with NaOH. The seal enhancer solution for increasing the probability of giga-seal formation contained (in mM): NaCl 80, KCl 3, MgCl_2_ 10, CaCl_2_ 35, and HEPES (Na^+^-salt) 10, adjusted to pH 7.4 with HCl. Data was acquired using PatchMaster v2x65 (HEKA Elektronik) and analyzed using Igor Pro 6.37. All experiments were performed at room temperature.

**The microelectrode arrays assay**

The extracellular field potential (FP) produced by hiPSC-CMs was measured by microelectrode array (MEA) assay using Multi Channel Systems MCS GmbH (Aspenhaustrasse, Reutlingen, Germany). The contracting areas of hiPSC-CMs were micro-dissected and plated on Matrigel® coated MEA chips containing 59 titanium electrodes and 1 internal reference electrode. Data was acquired for 5 minutes’ interval at baseline and post drug applications. The average beating frequency (BF), field potential duration (FPD) and Ca^2+^ wave were analyzed [7]. FPD was corrected by the beating frequency with Fridericia’s formula (FPDcF = FPD/interspike interval1/3) [8].

**References**

1. Yuill KH, Convery MK, Dooley PC, Doggrell SA, Hancox JC (2000) Effects of BDF 9198 on action potentials and ionic currents from guinea-pig isolated ventricular myocytes. Br J Pharmacol 130:1753-1766.
2. Ma D, Wei H, Zhao Y, Lu J, Li G, Sahib NB, et al. (2013) Modeling type 3 long QT syndrome with cardiomyocytes derived from patient-specific induced pluripotent stem cells. Int J Cardiol 168: 5277-5286.
3. Ma J, Guo L, Fiene SJ, Anson BD, Thomson JA, Kamp TJ, et al. (2011) High purity human-induced pluripotent stem cell-derived cardiomyocytes: electrophysiological properties of action potentials and ionic currents. Am J Physiol Heart Circ Physiol 301: H2006-2017.
4. Isenberg G, Klockner U (1982) Calcium tolerant ventricular myocytes prepared by preincubation in a “KB medium”. Pflueg Arch 395:6–18.
5. Amran MS, Hashimoto K, Homma N (2004) Effects of sodium-calcium exchange inhibitors, KB-R7943 and SEA0400, on aconitine-induced arrhythmias in guinea pigs in vivo, in vitro, and in computer simulation studies. J Pharmacol Exp Ther 310: 83-89.
6. Jin SS, Guo Q, Xu J, Yu P, Liu JH, Tang YQ (2015) Antiarrhythmic ionic mechanism of Guanfu base A-Selective inhibition of late sodium current in isolated ventricular myocytes from guinea pigs. Chin J Nat Med 13:361-367.
7. Rajamohan D, Kalra S, Duc Hoang M, George V, Staniforth A, Russell H, et al. (2016) Automated Electrophysiological and Pharmacological Evaluation of Human Pluripotent Stem Cell-Derived Cardiomyocytes. Stem Cells Dev 25: 439-452.
8. Clements M, Thomas N (2014) High-throughput multi-parameter profiling of electrophysiological drug effects in human embryonic stem cell derived cardiomyocytes using multi-electrode arrays. Toxicol Sci 140: 445-461.
